# Long‐term effects of widespread pharmaceutical pollution on trade‐offs between behavioural, life‐history and reproductive traits in fish

**DOI:** 10.1111/1365-2656.14152

**Published:** 2024-08-26

**Authors:** Upama Aich, Giovanni Polverino, Farin Yazdan Parast, Gabriela C. Melo, Hung Tan, James Howells, Reza Nosrati, Bob B. M. Wong

**Affiliations:** ^1^ School of Biological Sciences Monash University Clayton Victoria Australia; ^2^ Department of Ecological and Biological Sciences University of Tuscia Viterbo Italy; ^3^ Department of Mechanical and Aerospace Engineering Monash University Clayton Victoria Australia

**Keywords:** animal personality, antidepressants, Bayesian statistics, behavioural syndrome, chemical pollution, ecotoxicology, ejaculate traits, multivariate regression analysis

## Abstract

1. In our rapidly changing world, understanding how species respond to shifting conditions is of paramount importance. Pharmaceutical pollutants are widespread in aquatic ecosystems globally, yet their impacts on animal behaviour, life‐history and reproductive allocation remain poorly understood, especially in the context of intraspecific variation in ecologically important traits that facilitate species' adaptive capacities.
2. We test whether a widespread pharmaceutical pollutant, fluoxetine (Prozac), disrupts the trade‐off between individual‐level (co)variation in behavioural, life‐history and reproductive traits of freshwater fish.
3. We exposed the progeny of wild‐caught guppies (*Poecilia reticulata*) to three field‐relevant levels of fluoxetine (mean measured concentrations: 0, 31.5 and 316 ng/L) for 5 years, across multiple generations. We used 12 independent laboratory populations and repeatedly quantified activity and risk‐taking behaviour of male guppies, capturing both mean behaviours and variation within and between individuals across exposure treatments. We also measured key life‐history traits (body condition, coloration and gonopodium size) and assessed post‐copulatory sperm traits (sperm vitality, number and velocity) that are known to be under strong sexual selection in polyandrous species. Intraspecific (co)variation of these traits was analysed using a comprehensive, multivariate statistical approach.
4. Fluoxetine had a dose‐specific (mean) effect on the life‐history and sperm trait of guppies: low pollutant exposure altered male body condition and increased gonopodium size, but reduced sperm velocity. At the individual level, fluoxetine reduced the behavioural plasticity of guppies by eroding their within‐individual variation in both activity and risk‐taking behaviour. Fluoxetine also altered between‐individual correlations in pace‐of‐life syndrome traits: it triggered the emergence of correlations between behavioural and life‐history traits (e.g. activity and body condition) and between life‐history and sperm traits (e.g. gonopodium size and sperm vitality), but collapsed other between‐individual correlations (e.g. activity and gonopodium size).
5. Our results reveal that chronic exposure to global pollutants can affect phenotypic traits at both population and individual levels, and even alter individual‐level correlations among such traits in a dose‐specific manner. We discuss the need to integrate individual‐level analyses and test behaviour in association with life‐history and reproductive traits to fully understand how animals respond to human‐induced environmental change.

## Graphical abstract

Chronic exposure to antidepressants can impact non‐target animals, particularly the life‐history, behaviour and reproductive traits of male guppies. Key findings include lower sperm velocity, reduced behavioural plasticity and altered between‐individual correlations in pace‐of‐life syndrome traits, highlighting the need to better understand the ecological impacts of pollutants on wildlife.

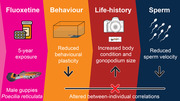

## 1 INTRODUCTION

Anthropogenic pollution is a pressing global challenge and is linked to significant biodiversity loss (Jaureguiberry et al., 2022). Evidence suggests that environmental pollutants can affect ecologically relevant traits, such as behaviour and life‐history (Dominoni et al., 2020; Saaristo et al., 2018), and alter species distribution and performance (Aulsebrook et al., 2020; Senzaki et al., 2020). For instance, exposure to pollutants can impact fundamental behaviours of wildlife—including activity, predator avoidance and reproductive behaviours—and disrupt the physiological functions of populations living in contaminated environments (reviewed in Aulsebrook et al., 2020; Bertram et al., 2022; Saaristo et al., 2018; Scott & Sloman, 2004). Moreover, long‐term exposure to pollutants can act as a potent selective force, driving the evolution of specific trait adaptations in wildlife. For example, the emergence of pesticide‐resistant traits in chronically exposed insect populations reveals the capacity of environmental pollutants to drive rapid evolutionary responses in wild organisms (Bras et al., 2022; Hawkins et al., 2019). Such responses can reshape the genetic diversity of a population, and potentially impact the long‐term viability of a population in a fast‐changing world (Whitehead et al., 2017). Yet conventional studies have predominantly focused on the average effects of pollutants on behavioural and life‐history traits of the animals, while neglecting variation at the individual level (reviewed in Montiglio & Royauté, 2014), even though such variation is known to have important ecological and evolutionary consequences (Réale et al., 2007; Wolf & Weissing, 2012).

Evidence from the handful of studies that have examined variation at the individual level reveals that the effects of environmental pollutants often differ between and within individuals of a population, resulting in a mosaic of outcomes that are likely to impact its resilience to future challenges (Polverino et al., 2021). For example, exposure to pollutants has been reported to largely reduce behavioural repeatability (i.e. the proportion of behavioural variance observed within and between individuals of a population) in jumping spiders (Royauté et al., 2015) and freshwater fish (Tan et al., 2020). More recently, pollutants have also been shown to drive such overall decline in behavioural variation by selectively suppressing behavioural differences between (sensu behavioural individuality: Polverino et al., 2021, 2023; Réale et al., 2007) and within individuals (sensu behavioural plasticity: Dingemanse & Wolf, 2013; Henry et al., 2022; Polverino et al., 2023). As a target of selection, individual‐level, and especially between‐individual variation in ecologically relevant traits plays a key role in shaping ecological and evolutionary processes (Wolf & Weissing, 2012). For instance, greater behavioural variation between individuals was found to benefit both the population growth and longevity in ant (*Temnothorax longispinosus*; Modlmeier et al., 2012) and salmonid populations (*Oncorhynchus tshawytscha*; Carlson & Satterthwaite, 2011) in the face of environmental challenges. On the contrary, lower behavioural variation between individuals increased the risk of extinction in populations of wild sockeye salmon (*Oncorhynchus nerka*; Schindler et al., 2010). As such, accounting for individual‐level responses is critical if we are to achieve a proper understanding of the effects of pollutants on the ecological dynamics and evolutionary trajectories of animal populations.

Theory predicts that certain traits should be closely associated with one another, such that individuals within a population will typically differ between each other in a suite of correlated traits (Réale et al., 2010; Sih et al., 2015; Wolf et al., 2007; Wolf & Weissing, 2010). Therefore, between‐individual differences in behaviour should be integrated with morphological and life‐history variation along a continuum of pace‐of‐life syndrome (POLS) strategies, in which individuals differ from each other in the amount of resources allocated to either survival or reproduction (Réale et al., 2010). For instance, bolder individuals may take more risk to secure more resources and thus gain larger size at maturity (Kern et al., 2016), better body condition (Biro & Stamps, 2008) and a higher reproductive value (Gasparini, Speechley, & Polverino, 2019), but at the cost of a less efficient immune system (Woodhams et al., 2016) and a shorter lifespan (Careau et al., 2010). Yet recent comparative analyses suggest high heterogeneity in the overall support for the POLS hypothesis, with substantial variation in the effect size and even in the direction of the correlations observed among POLS traits across empirical studies (Chang et al., 2024; Laskowski et al., 2021; Royauté et al., 2018). In fact, it is reasonable to assume that ecological conditions might not only shape specific life‐history, physiological and behavioural traits, but correlations between the entire suite of traits might be adaptively modulated by the environment (extended pace‐of‐life syndrome hypothesis; Dammhahn et al., 2018, and references therein). In line with this perspective, recent studies have found that the coevolution of correlated traits depends upon specific selective processes, such as predator regimes (Dhellemmes et al., 2021; Polverino et al., 2018), with selection favouring particular locally adaptive suites of correlated traits at the between‐individual level (Godin et al., 2022). If environmental stressors can favour or obliterate individual‐level correlations between pace‐of‐life syndrome traits (Dammhahn et al., 2018; Hämäläinen et al., 2021), it is reasonable to expect that correlations between behaviour, life‐history and reproductive traits might also vary depending on the presence (and concentration) of disruptive pollutants in the wild. Yet such impacts of environmental pollutants remain largely untested. Understanding whether and how pollution disrupts the relationships between behaviour, life‐history and reproduction of animals is critical for assessing the impacts of global pollutants on the ecology and evolution of wildlife, and predicting how populations will respond to future changes. If global pollutants alter the trade‐offs between traits, genetic correlations might no longer be adaptive for animals living in contaminated environments, potentially affecting the evolution of those traits over generations (Killen et al., 2013).

Here, we tested whether multigenerational exposure to a globally significant pharmaceutical pollutant, fluoxetine (Prozac), disrupts individual‐level (co)variation between behavioural, life‐history and reproductive traits in fish. Fluoxetine is a widely prescribed antidepressant and pharmaceutical pollutant detected in aquatic environments worldwide, with average concentrations in surface waters ranging from <1 to 350 ng/L (Christensen et al., 2009; Kolpin et al., 2002; Webb, 2001). We exposed the progeny of wild‐caught guppies (*Poecilia reticulata*) to field‐relevant levels of fluoxetine for 5 years (corresponding up to 15 generations). We then quantified the behaviour of adult males repeatedly over time (activity and risk‐taking) and measured their life‐history (body condition, gonopodium size and body coloration) and post‐copulatory reproductive traits (sperm vitality, number and velocity) that are known to be under strong sexual selection in species where females mate with multiple males (Fitzpatrick & Lüpold, 2014; Gasparini, Devigili, & Pilastro, 2019). We focus on the effects on male guppies for several reasons. First, previous research has shown that male behavioural, life‐history and reproductive traits may be especially sensitive to selection pressures associated with changes to their environment, including changes of anthropogenic origin, such as chemical pollution (Bertram et al., 2018; Cole & Endler, 2015; Reznick & Bryga, 1987). Second, studies have suggested that between‐individual correlations between behavioural and life‐history traits should be stronger in males than in females, since gamete production is typically less costly for males and, therefore, male fitness is often limited by access to mates and fertilisation (see Hämäläinen et al., 2018 and references therein). We used a multivariate statistical approach that partitions mean effects and effects between‐ and within‐individuals to test whether global pollutants altered the between‐individual (co)variation between these traits. We predict that long‐term exposure to fluoxetine would impact the (co)variation between behavioural, life‐history and reproductive traits in fish, as predicted by the extended pace‐of‐life syndrome hypothesis (Dammhahn et al., 2018) and in line with previous work showing a reduction in behavioural variation induced by fluoxetine (Polverino et al., 2021, 2023). It is conceivable that fluoxetine might strengthen the association between individual behaviour, life‐history and reproductive traits along a fast‐slow pace‐of‐life continuum, since behavioural alignment with environmental conditions can be crucial for optimising the life‐history pace (Hämäläinen et al., 2018). However, empirical studies that tested trait covariation under environmental contamination are very rare and, when present, reported results inconsistent with general predictions (Debecker & Stoks, 2019), suggesting that a comprehensive statistical approach is needed to shed light on this important question.

## 2 METHODS

### 2.1 Multigenerational exposure

Sexually mature wild guppies (*n* = 3600) were collected from Alligator Creek (19°23′50.3′′ S, 146°56′56.5′′ E), Townsville, Australia to establish a long‐term mesocosm system (Tan et al., 2020) and to test for the multigenerational, chronic effect of pharmaceutical pollution on freshwater fish. Adult fish (*n* = 300; 50:50 sex ratio) were randomly assigned to one of 12 independent mesocosms (180 × 60 × 60 cm; water depth: 30 cm; 648 L) and housed in a temperature‐controlled facility (23.4 ± 1.0°C) under a 12:12 h light:dark cycle. Our indoor mesocosm tanks (sensu Gotanda et al., 2019; Sommer et al., 2007; Wood et al., 2022) contained carbon‐filtered freshwater and were aerated by commercial air pumps (Resun LP100). Water quality was maintained through partial water changes in which ~20% of the water from each mesocosm was replaced weekly. Each mesocosm contained a 3 cm layer of gravel (7 mm grain size), rocks and aquatic plants (*Taxiphyllum barbieri*) to simulate the guppies' natural habitat. To supplement the diet (sensu Gotanda et al., 2019; Wood et al., 2022) of the largely self‐sustaining populations of guppies in our set‐up, commercial fish food (Aquasonic Nutra Xtreme C1) was offered to the animals every second day.

**FIGURE 1:**
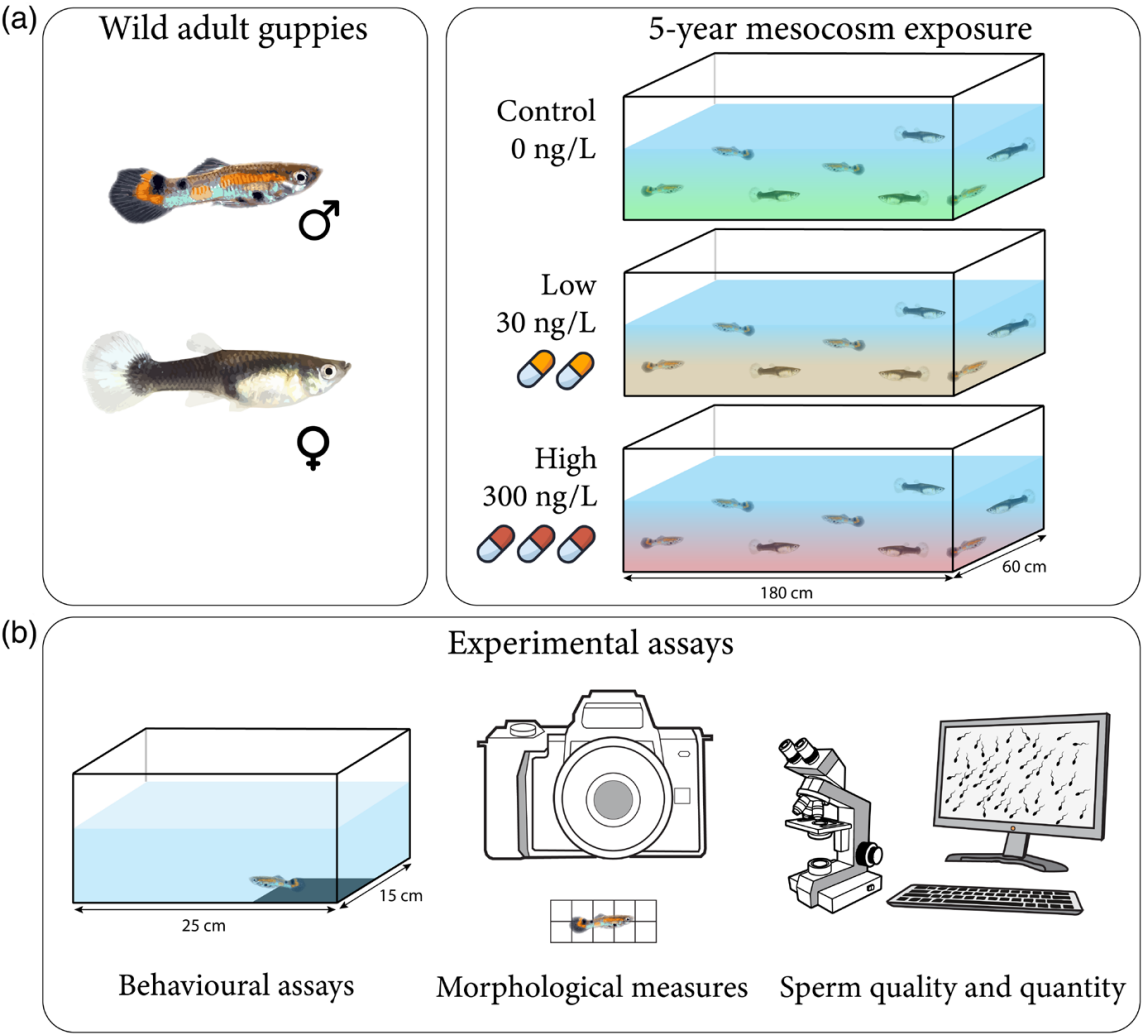
Schematic of the exposure protocol and experimental design. (a) Adult guppies with an equal sex ratio were collected from the wild and exposed to control, low and high fluoxetine concentrations across 12 replicated mesocosm populations (180 × 60 × 60 cm) for five consecutive years. (b) Following exposure, males from each treatment (four independent mesocosms per treatment) were assayed for their behavioural, life‐history and sperm traits.

After 5 months of acclimation to the mesocosms, fish were exposed to fluoxetine. Full details of mesocosm establishment and fluoxetine exposure have been reported elsewhere (Polverino et al., 2021; Tan et al., 2020). Briefly, fish were randomly assigned to one of three exposure treatments (*n* = 4 independent mesocosm populations per treatment): control (no fluoxetine), low fluoxetine (31.5 ± 2 ng/L) and high fluoxetine (316 ± 18.45 ng/L) for five consecutive years (i.e. 60 months; Figure 1). The low‐ and high‐fluoxetine treatments reflect concentrations repeatedly detected in freshwater habitats, with the former representing common surface water concentrations in fluoxetine‐contaminated systems and the latter representing levels typically found in heavily effluent‐dominated waterbodies (Mole & Brooks, 2019). The treatment concentrations of fluoxetine in the mesocosms (verification using HPLC‐MS–MS) are summarised in Table S1. Experiments complied with all relevant State and Federal Laws of Australia and were approved by the School of Biological Sciences Animal Ethics Committee (approval numbers 14787, 25541, 31120).

### 2.2 Experimental fish

We assayed the behaviour, morphology and sperm traits of 144 sexually mature males (12 individuals per mesocosm, 48 per treatment). Adult males were randomly captured from their population mesocosms 4 months after we cessated supplementing the pollutant and transferred to individual glass housing tanks (12 × 23 cm, diameter × height) filled with 2 L of treatment water from their native mesocosm. We used mesocosm water for housing and behavioural assays, as fluoxetine is pseudo‐persistent in nature and continues to exert its effects on the aquatic environment even after the cessation of direct input (Kwon & Armbrust, 2006), potentially influencing the physiological and behavioural responses of fish. Each housing tank contained a gravel substrate (2 cm layer) and live vegetation (Java moss). Water temperature (24 ± 1.0°C) was monitored daily, and fish were kept on a 12:12 h light:dark cycle. Fish were fed until satiation as in their native mesocosms and were acclimated to the individual holding tanks for 72 h before behavioural assays commenced.

### 2.3 Behavioural assays

We ran an open‐field assay validated for studying individual‐level variation in the behaviour of guppies (Burns, 2008). Before a trial started, a fish was placed into an opaque plastic cylinder and allowed to acclimate for 5 min. Individual fish were then carefully placed into an open‐field arena (25 × 15 × 15 cm), which contained a white background with a dark, squared region (7.5 × 7.5 cm) in one corner that served as a refuge (Figure 1b). The presence of the refuge ensured that fish had a safe area accessible when they chose to explore the novel and potentially more ‘risky’ open space. The arenas were filled with water from the respective mesocosms to ensure that exposure to treatment water was maintained for each fish throughout the experiment. Water was replaced in the arenas between consecutive trials to eliminate the potential build‐up of conspecific cues.

Upon entering the arena, a fish was allowed to explore the novel space freely for 15 min, and its behaviour was filmed from above with a high‐resolution camera (Panasonic HC‐V180). Following the completion of the trial, the fish was gently netted and returned to its individual housing tank. This process was repeated three times for each fish (i.e. three repeated measures per individual), with 2‐day intervals between consecutive trials. Fish were tested in a random order, and experimental videos were automatically tracked using EthoVision XT v. 14.0.1326 (Noldus Information Technologies), blind to treatment.

We selected fish activity (the total distance moved by an individual in centimetre) and risk‐taking (refuge use; use of the refuge in seconds, and consequently not exploring open spaces that are unfamiliar and potentially dangerous) as the specific biologically relevant traits we were interested in this study. Individual variation in these traits is a key target of selection in all non‐sessile animals (Réale et al., 2007) and is known to have ecological and evolutionary implications (Wolf & Weissing, 2012).

After the behaviour assays, males were returned to their individual tanks for 5 days to standardise their social and sexual experience before we measured their morphology and sperm traits.

### 2.4 Morphological traits

Males were anaesthetised in a water bath with clove oil before measuring their colour patterns and body size. Individuals were photographed (Nikon D5100, 60 mm lens) at 90° to the right lateral plane with standardised lighting and camera conditions across photographs. Soon after, fish body mass was recorded using a digital analytical scale (Scientech ZSA 210 scale; ±0.0001 g). We used ImageJ software to measure fish standard body length (snout to caudal peduncle) and gonopodium length (base of gonopodium to distal tip). We then calculated a scaled mass index (SMI) for each fish, as a proxy for its body condition. To do so, we log‐transformed fish body weight and length to produce a *β* coefficient (*β* = 3.203), which was used with the mean body length of fish (mean body length = 17.43 mm) to estimate the SMI value at an individual level following Peig and Green (2009). We used Adobe Photoshop CC (version 24.2) selection tools such as the Lasso Tool and its derivatives and the Photoshop measurement feature (measurement log) to estimate individual male body area (including the caudal fin) and colour patterns: orange area (surface area of orange, yellow and red patches) and black area (encompassing black and fuzzy black patches), as per Gasparini et al. (2010). All body coloration measures were collected blind to the treatment. Female guppies are known to assess and respond to overall or composite male coloration through mate choice, with both orange and black coloration being preferred in Australian and Trinidadian guppies (see Brooks & Endler, 2001a, 2001b; Houde, 2019). Therefore, the proportion of total coloration, after accounting for body size, was used in our analyses, following Godin et al. (2022).

### 2.5 Sperm traits

After the morphological measures, each male was placed under a dissecting microscope on a glass slide. To release the ejaculate of the fish, a metal probe was used to swing the gonopodium forward, and a gentle pressure was applied to the abdomen. The fish was then covered with 100 μL of extender medium (207 mM NaCl, 5.4 mM KCl, 1.3 mM CaCl_2_, 0.49 mM MgCl_2_, 0.41 mM MgSO_4_, 10 mM Tris and pH 7.5) to render the sperm inactive.

For sperm velocity assays, two samples of five sperm bundles were extracted from each individual and transferred to a microtube containing 5 μL of extender medium. Each sample was then added to 20 μL of activation solution (150 mM KCl and 2 mg/mL bovine serum albumin) and 0.3 μL of a diluted fluorescent dye (0.1 SYBR 14:5 activator medium) to stain the live cells. The 25.3 μL solution was gently resuspended ~100 times to separate sperm bundles. We then took 5 μL aliquots from the solution, gently placed them in a cell of a 12‐cell multi‐slide, added a coverslip and placed it on an IX83 inverted microscope equipped with an ORCA‐Flash4.0V3 Digital CMOS camera. We used the microscope in fluorescent mode to record a 5‐s video at 30 frames per second for sperm motility analysis. Sperm motility parameters, including average path velocity (VAP), curvilinear velocity (VCL), straight‐line velocity (VSL) and linearity (LIN), were evaluated with the OpenCASA (Open Source Computer Aided Sperm Analysis) plugin in ImageJ (Alquézar‐Baeta et al., 2019). We analysed VCL to allow a broader comparison of sperm velocity across fish species (see Aich et al., 2022; Gasparini, Devigili, & Pilastro, 2019). The CASA settings were set to those of previously reported studies using the same system (Vasilescu et al., 2023). We took two repeated measures per sample. Sperm velocity for each male was calculated as the average of the two samples, accounting for the number of sperm tracked per sample, following standard protocols (Aich et al., 2022). An average of 65 sperm was tracked and analysed in each recorded video.

For sperm number and vitality measures, the remainder of the ejaculate was transferred to a 1.5 mL Eppendorf tube containing 100–900 μL of extender medium, with the quantity of extender medium used depending on the amount of ejaculate released by each male. We gently resuspended and vortexed the ejaculate contents of the 1.5 mL Eppendorf tube for 1 min to break up sperm bundles and distribute the sperm evenly throughout the sample. The sperm was then stained using the LIVE/DEAD Sperm Vitality Kit (L‐7011; Thermo Fisher) and 20 μL of the sample was pipetted into a haemocytometer counting chamber. An IX83 inverted fluorescence microscope (Olympus, Japan) equipped with an ORCA‐Flash4.0V3 Digital CMOS camera (Hamamatsu, Japan) was used to capture green (live cells) and red (dead cells) fluorescence images with a 10× magnification objective. An average of 51 sperm was counted per sub‐chamber, depending on varying diluting factors. The images were then analysed in ImageJ to quantify sperm number (based on the total number of live and dead sperm, averaged by three subsamples) and vitality (ratio of live to the total sperm number), blind to the treatment.

### 2.6 Statistical analyses

Of the 144 fish, 139 individuals completed all behavioural and life‐history trials (*n* = 45 control, 46 low and 48 high), and 134 had sperm to measure (*n* = 45 control, 43 low and 46 high). We analysed the data using a Bayesian multivariate mixed‐effects model fitted with the *brms* package (Bürkner, 2017) in *R* v. 4.2.3 (R Core Team, 2023).

The model included two behavioural traits (activity and refuge use), three life‐history traits (body condition, coloration and gonopodium size) and three sperm traits (sperm vitality, velocity and count) as the response variables. Treatment was included as a fixed factor, while trial (three repeated measures per individual) was included as a covariate for behavioural traits only. All response variables were mean‐centred (i.e. mean = 0; SD = 1), and trial was left‐centred (i.e. trial 1 = 0, to set the model intercept at the first trial) prior to the analysis to aid in model fitting and interpretation. Mesocosm (*n* = 12; four per treatment) was included as a random factor in our model to account for the variables associated with housing conditions. Male ID per treatment was also included as a random intercept in the model to estimate the extent of the between‐individual variance across treatments. For behavioural traits, the residual portion of the model allowed us to estimate the within‐individual (residual) variance across treatments—while we set the residual variance to a small reference value (0.01) for life‐history and sperm traits, which had one data point per individual (Houslay & Wilson, 2017). Further, we calculated the magnitude difference (Δ*V*) in between‐individual variance (ΔVA) for all traits and within‐individual (ΔVW) variance for behavioural traits only (Royauté & Dochtermann, 2021) to directly measure the distribution of Δ*V*s by estimating the difference in the posterior distributions of two separate variance components. The model further allowed us to examine whether between‐individual correlations (pace‐of‐life‐syndrome) between traits differed across treatments. Following McElreath (2020), we report posterior means with both 89% and 95% credible intervals (instead of the commonly used 95% CI) in the Supporting Information to discourage readers from conducting unconscious hypothesis testing. However, our inference is based on non‐overlapping 89% CIs with zero, as these are deemed more stable than the equally arbitrary 95% level (Kruschke, 2015). That is, clear evidence for an effect was considered when CIs did not include zero (Royauté & Dochtermann, 2021). See Table S2 for full model output.

The multivariate model ran for 8000 iterations (3000 warmups) on four chains (thinning interval = 2) using weakly informative priors (Lemoine, 2019). We also ran the model using default priors, as recommended by Dingemanse and Dochtermann (2013) and verified that results were consistent and independent of the priors used (Table S3). Posterior density plots were checked to ensure proper model mixing, with the model converging with low among‐chain variability (all Rhat = 1). *R* code with full model syntax is provided as Supporting Information.

## 3 RESULTS

### 3.1 Mean‐level effects

#### 3.1.1 Behavioural traits

We found that low fluoxetine exposure had a weak effect on the average activity and refuge use of the fish, with CIs marginally overlapping with zero, indicating some uncertainty around this evidence (Table S2). In particular, males from the low fluoxetine treatment tended to be less active (estimate ± 89% CI: −0.28 [−0.6, 0.04]) and spent more time in the refuge than unexposed fish (estimate ± 89% CI: 0.26 [−0.04, 0.55])—indicating lower activity and higher refuge use, respectively. Such effects were absent in fish from the high fluoxetine treatment (Table S2). Fish decreased their average activity as trials progressed (estimate ± 89% CI: −0.22 [−0.29, −0.16]). On the contrary, trial number had positive effects on refuge use, with an increase in refuge use over successive trials (estimate ± 89% CI: 0.19 [0.11, 0.27]; Table S2).

#### 3.1.2 Life‐history traits

**FIGURE 2:**
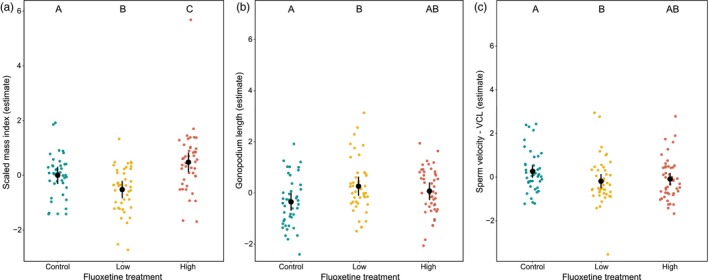
Mean‐level effect of fluoxetine on male (a) body condition (scaled mass index, in g), (b) gonopodium length (mm) and (c) sperm velocity (VCL, μm/s). The letters denote significant differences between treatments. In each plot, filled‐black circles represent the mean estimates, vertical error bars denote 89% credible intervals and coloured‐dotted values represent the probability densities of conditional effects.

Fluoxetine exposure had a non‐monotonic effect on body condition (Table S2; Figure 2a): males from the low fluoxetine treatment had a lower body condition, and males from the high fluoxetine treatment had a higher body condition than control fish (estimates ± 89%CI: −0.52 [−0.976, −0.068]; 0.48 [0.01, 0.96]). Fluoxetine exposure also had a positive effect on male gonopodium size, with males from both low and high treatment having longer gonopodia compared to control males (estimate ± 89% CI: 0.61 [0.09, 1.12]; 0.42 [−0.08, 0.91]), although CIs marginally overlapping with zero in the high treatment indicated some uncertainty around this evidence (Figure 2b). In contrast, fluoxetine exposure did not affect the proportion of body coloration (Table S2).

#### 3.1.3 Sperm traits

Exposure to fluoxetine had no detectable effects on sperm viability and count (Table S2). On the contrary, fluoxetine had a negative effect on male sperm velocity: fish from both low and high fluoxetine treatment had a lower sperm velocity compared to control males (estimates ± 89% CI: −0.45 [−0.88, −0.02]; −0.35 [−0.76, 0.05]; Figure 2c) with CIs marginally overlapping with zero in the high treatment.

### 3.2 Variation between and within individuals

#### 3.2.1 Behavioural traits

**FIGURE 3:**
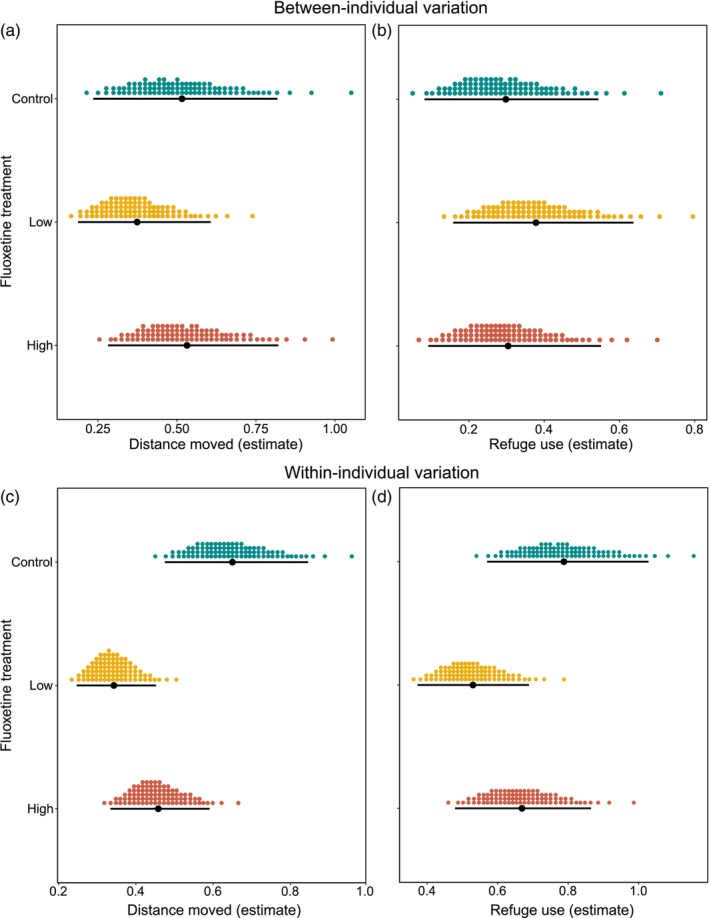
Between‐individual variance in (a) activity (distance moved, in centimetre) and (b) refuge use (in seconds) and within‐individual variance in (c) activity (distance moved, in centimetre) and (d) refuge use (in seconds) of males from the three exposure treatments (control, low fluoxetine and high fluoxetine). In each plot, filled‐black circles represent the mean–variance estimates, vertical error bars denote 89% credible intervals and coloured‐dotted values represent probability densities.

We found no evidence that exposure to fluoxetine affected between‐individual variation in the behaviour of our fish. Specifically, the between‐individual variance in both activity and refuge use did not vary between males from the three exposure treatments (Table S4; Figure 3a,b). In sharp contrast, behavioural plasticity (i.e. residual within‐individual variation) was higher in control than in fluoxetine‐exposed males (Table S5): control males had higher levels of within‐individual variation in their activity than males from both the low and high fluoxetine treatment (ΔVW [89% CI]: 0.307 [0.124, 0.48]; 0.193 [0.004, 0.375], respectively). We found weak evidence that males from high‐exposure treatment had a higher within‐individual variation in activity than low‐exposure males, with CI's slightly overlapping zero ((ΔVW [89% CI]: 0.114 [−0.025, 0.252]); Figure 3c). For refuge use, control males also exhibited higher within‐individual variance than low fluoxetine‐exposed males (ΔVW [89% CI]: 0.259 [0.022, 0.486]), but control and high‐treatment males did not differ in their plasticity levels (ΔVW [89% CI]: 0.119 [−0.147, 0.375]). We again found weak evidence that males from high‐exposure treatment had a higher within‐individual variation in their refuge use than low‐exposure males with CI's slightly overlapping zero (ΔVW [89% CI]: 0.14 [−0.063, 0.358]; Figure 3d).

#### 3.2.2 Life‐history traits

**FIGURE 4:**
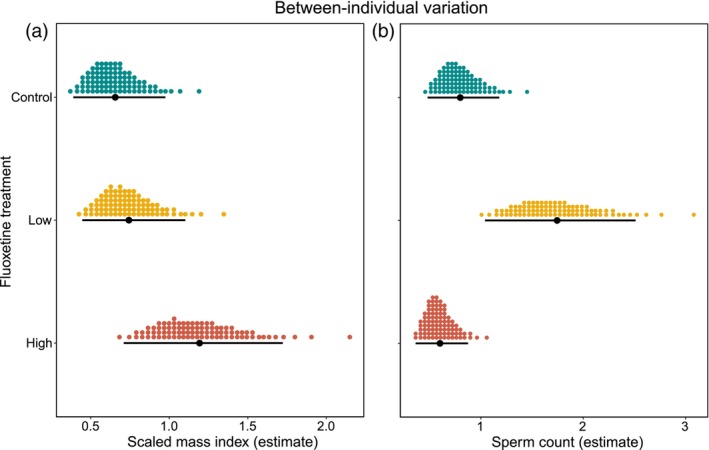
Between‐individual variance in (a) body condition (scaled mass index) and (b) sperm count of males from the three exposure treatments (control, low fluoxetine and high fluoxetine). In each plot, filled‐black circles represent the mean–variance estimates, vertical error bars denote 89% credible intervals and coloured‐dotted values represent probability densities.

Fish exposed to high fluoxetine concentrations showed the highest level of between‐individual variation in body condition: high fluoxetine males differed more from each other in their body condition than control (ΔVA [89% CI]: −0.537 [−1.025, −0.072]; Figure 4a) and low fluoxetine males (ΔVA [89% CI]: 0.45 [−0.028, 0.966]; Figure 4a), although credible intervals slightly overlapped with zero. Yet body condition did not differ at the between‐individual level between control and low treatment males (ΔVA [89% CI]: −0.09 [−0.452, 0.288]). In contrast, fluoxetine exposure had no detectable effect on the between‐individual variance observed in both gonopodium size and body coloration across treatments (Table S4).

#### 3.2.3 Sperm traits

Fluoxetine exposure had no detectable effect on the between‐individual variance observed in both sperm velocity and sperm viability (Table S4). In contrast, both control and high‐treatment males had lower between‐individual variation in their sperm number than males from the low fluoxetine treatment (ΔVA [89% CI]: −0.945 [−1.576, −0.232]; −1.143 [−1.771, −0.503], respectively; Figure 4b).

### 3.3 Between‐individual correlations in traits

#### 3.3.1 Behaviour

We found that activity and refuge use were negatively correlated at the between‐individual level across treatments (Figure S1; Table S6): control (*r* [89% CI]: −0.431 [−0.643, −0.181]), low (*r* [89% CI]: −0.367 [0.576, −0.127]) and high fluoxetine treatment (*r* [89% CI]: −0.357 [−0.570, −0.117]). In other words, fish that swam more also hid the least, independently of the exposure treatment.

#### 3.3.2 Life‐history traits

We found weak evidence of a negative between‐individual correlation between body condition and coloration in the high fluoxetine treatment; males in better body conditions tended to be less coloured than others when living in highly polluted waters (*r* [89% CI]: −0.171 [−0.355, −0.024]). While low fluoxetine males that were more colourful had smaller gonopodia than less coloured individuals (*r* [89% CI]: −0.142 [−0.325, −0.058]; Figure S1; Table S6).

#### 3.3.3 Sperm traits

Sperm traits were generally not correlated at the between‐individual level across treatments (Figure S1; Table S6). Except in low‐treatment fish, individuals with more sperm had lower sperm vitality than males with less sperm (*r* [89% CI]: −0.212 [−0.393, −0.010]). We found a weak positive correlation in sperm count and velocity in fish from high fluoxetine treatment (*r* [89% CI]: 0.128 [−0.073, 0.317]) with CI's overlapping zero.

#### 3.3.4 Pace‐of‐life syndrome

**FIGURE 5:**
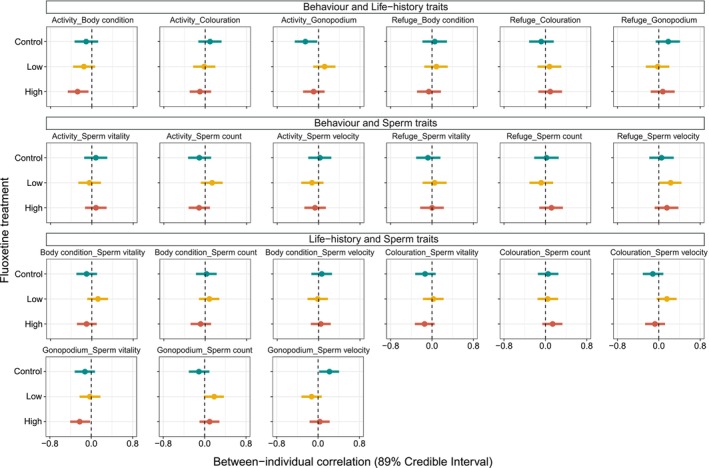
Between‐individual correlation in behaviour, life‐history, and sperm traits (i.e. pace‐of‐life syndrome) of males. In each plot, filled circles with horizontal error bars denote correlational values 89% credible intervals, and the colours represent three exposure treatments (control, low fluoxetine and high fluoxetine). The black dashed line indicates no correlation effects, while the values on the right side of the line show a positive relationship, and the ones on the left side indicate a negative relationship between traits.

Behavioural, life‐history and sperm traits were often correlated at the between‐individual level (i.e. pace‐of‐life syndrome), but correlations often varied in sign and strength across treatments (Figure 5; Table S6).

When looking at behavioural and life‐history traits, we found a negative correlation between activity and gonopodium size in control males (*r* [89% CI]: −0.256 [−0.461, −0.027]) and a weak, positive correlation between refuge use and gonopodium size, with CI's slightly overlapping zero (*r* [89% CI]: 0.183 [−0.059, 0.408]). In other words, control fish with a longer gonopodium swam on average less and hid more than others. We also found a negative correlation between activity and body condition in both high‐treatment (*r* [89% CI]: −0.276 [−0.464, −0.065]) and low‐treatment fish (*r* [89% CI]: −0.154 [−0.362, 0.063]); fluoxetine‐exposed fish with better body condition swam on average less than others.

Instead, low and high fluoxetine concentrations had opposite effects on the correlation between activity and sperm number: low fluoxetine males that were more active also had a higher sperm count (*r* [89% CI]: 0.134[−0.085, 0.340]), while the sperm count was lower in very active fish exposed to high fluoxetine levels (*r* [89% CI]: −0.119 [−0.321, 0.091]), with both CIs overlapping zero. We also detected a weak negative correlation between activity and sperm velocity in low fluoxetine males (*r* [89% CI]: −0.128 [−0.336, 0.094]), meaning that individuals that swam more had slower sperm, which was not observed in males from the control and the high‐fluoxetine treatment (Table S6). A positive correlation between refuge use and sperm velocity was detected in both the low (*r* [89% CI]: 0.229 [0.002, 0.439]) and high‐treatment fish (*r* [89% CI]: 0.156 [−0.08, 0.3788]); i.e. fluoxetine‐exposed fish with higher sperm velocity spent more time in refuge than control males.

In the case of life‐history and sperm traits, gonopodium size and sperm vitality were negatively correlated in high fluoxetine (*r* [89% CI]: −0.224 [−0.407, −0.024]) and control males with CIs slightly overlapping zero (*r* [89% CI]: −0.122 [−0.319, 0.072]), but not in low fluoxetine males (*r* [89% CI]: −0.03 [−0.226, 0.176]). In other words, males with a longer gonopodium had less viable sperm in the control and high fluoxetine treatments, but this pattern was not detected in low fluoxetine males. We found a positive correlation between gonopodium size and sperm velocity in control males (*r* [89% CI]: 0.22 [0.02, 0.406]), but this correlation was negative in low fluoxetine males (*r* [89% CI]: −0.126 [−0.321, 0.071]) and absent in high fluoxetine males (*r* [89% CI]: 0.03 [−0.163, 0.224]), indicating that males with longer gonopodium had higher sperm velocity only in the control treatment, while this pattern was disrupted or reversed in fish exposed to fluoxetine. The complete list of pairwise correlations is reported in Table S6.

## 4 DISCUSSION

The global increase in pharmaceutical pollutants in aquatic environments is an emerging ecological concern. However, the extent to which these pollutants may disrupt associations in the between‐individual variation in animal behaviour, life‐history and reproductive traits remains poorly understood. We found evidence that fluoxetine has mean effects on fish behaviour, life‐history and sperm traits: low fluoxetine exposure (31.5 ng/L) reduced activity and increased refuge use in male guppies, and also resulted in altered body condition, increased gonopodium size and reduced sperm velocity. At the individual level, both low and high concentrations of fluoxetine (31.5 and 316 ng/L, respectively) reduced behavioural plasticity of fish, suppressing the within‐individual variation in both activity and refuge use of guppies exposed to the pollutant. On the contrary, behavioural variation between individuals did not generally vary across treatments, although we detected strong dose‐specific effects on between‐individual variation in both body condition and sperm number. Importantly, fluoxetine exposure resulted in the emergence of pace‐of‐life syndrome correlations between behavioural and life‐history traits (e.g. activity and body condition) and between life‐history and sperm traits (e.g. gonopodium size and sperm vitality), but it disrupted correlation between behaviour and other life‐history traits (e.g. activity and gonopodium size). Our findings reveal that the long‐term exposure to a widespread pharmaceutical pollutant can disrupt the trade‐offs between behaviour, life‐history and reproductive traits in guppies, indicating that population resilience and individual adaptive potential in wildlife can be compromised in contaminated habitats.

### 4.1 Mean level effects

We found that body condition deteriorated in fish exposed to low fluoxetine concentration, but improved in fish exposed to high concentration, compared to control males. This result is partially in line with previous findings where lower concentrations of this pollutant compromised body condition in mosquitofish (42 ng/L: Bertram et al., 2018). Such non‐monotonic, dose–response relationships are well reported in the literature (see Tan et al., 2023 and references therein). They could result from several different mechanisms, including receptor desensitisation, negative feedback with increasing dose, dose‐dependent metabolism modulation and/or opposing effects induced by an analyte binding to multiple receptors that differ in their affinity (Vandenberg et al., 2012). Body condition is an important mediator of sexual selection in adult guppies for male–male competition, courtship behaviour and coercive copulation with females (Godin et al., 2022). Therefore, the dose‐dependent effects observed here on body condition may lead to varying fitness outcomes in male guppies exposed to different polluted environments. For instance, costs associated with phenotypic adaptations to contaminated waterways could potentially reduce the fitness of such animals if pollution levels decrease in the environment (Hamilton et al., 2017).

We also found an increase in gonopodium size following the multigenerational exposure to fluoxetine, although such an effect was not found following acute exposure to the pollutant in mosquitofish (42 ng/L: Bertram et al., 2018). Since a longer gonopodium is beneficial for coercive mating in guppies (Evans et al., 2011) and fluoxetine exposure can increase male coercive mating behaviour (350 ng/L: Fursdon et al., 2019; 38 and 312 ng/L: Wiles et al., 2020), selection for longer gonopodia can favour males to gain higher pre‐copulatory mating success (Head et al., 2015). This should be especially important for males living in polluted waters if the exposure to the pollutant has negative effects on male post‐copulatory traits—that is, it compromises the competitiveness of sperm and reduces fertilisation success.

We found that fluoxetine exposure resulted in a reduction in sperm velocity, yielding similar results to those reported in humans (reviewed in Beeder & Samplaski, 2020). Likewise, in line with previous findings, we found no effect of the pollutant on sperm vitality (guppies: Martin et al., 2019). Although acute exposure to low fluoxetine has been reported to increase sperm number in mosquitofish (42 and 380 ng/L: Bertram et al., 2018), we did not find such an effect in guppies. However, the lack of an average effect in sperm number should not be interpreted as a lack of change but rather as a greater variation between individuals following fluoxetine treatment (see Section 4.2 and Sanderson et al., 2023).

Finally, we found a weak effect on behaviour where exposure to low fluoxetine concentrations reduced fish activity and risk‐taking behaviour (i.e. increased refuge use). Similar findings have been reported in guppies when acute exposure to the pollutant reduced activity (500 ng/L: Saaristo et al., 2017) and long‐term exposure decreased risk‐taking behaviour (40 and 366 ng/L: Polverino et al., 2021). However, it is important to note that the average effects of fluoxetine on behavioural traits are not always consistent across species and can vary depending on the exposure period and dosage (reviewed in Correia et al., 2023; Salahinejad et al., 2022). These findings underscore the complexity of differential responses at the population level, where fluoxetine induces dose and trait‐specific effects with potential consequences for the ecological dynamics of wildlife.

### 4.2 Individual‐level variation in behaviour, life‐history and sperm traits

In contrast to previous findings, we found that fluoxetine did not impact behavioural differences between individuals (i.e. behavioural individuality). This is likely due to temporal dynamics in behavioural variation related to the duration of the exposure that largely differed between the 2‐year exposure in Polverino et al., (40 and 366 ng/L: 2021, 2023) and the 5‐year exposure in this work. This might suggest populations adaptively responding to the continued presence of pollution over many generations, as observed in both freshwater killifish (Whitehead et al., 2017) and snails (32.7 ng/L: Henry et al., 2022). Although the exact mechanism behind this finding is unknown, we suspect that as the exposure continues through multiple generations, individuals might develop behavioural resistance to the effects of fluoxetine, which, in turn, could lead to a restoration of between‐individual variation in behaviour at the population level (Sih, 2013). Understanding the proximate mechanism driving the observed changes in behavioural individuality is warranted in future studies.

On the contrary, we found a strong, non‐monotonic effect of fluoxetine on the behavioural plasticity of males. The reduction in the (residual) within‐individual variation in both activity and refuge use suggests a potential stabilising effect of fluoxetine on the behaviour of individuals over time. Similar effects of fluoxetine have been found in both snails and female guppies, in which long‐term exposures reduced within‐individual variance in their behaviour (32.7 ng/L: Henry et al., 2022; 40 and 366 ng/L: Polverino et al., 2023). Such a stabilising effect of fluoxetine on behavioural plasticity might be driven by selective pressures favouring males with more consistent behavioural profiles. If the consistency of individual responses is a target of sexual selection (Adriaenssens & Johnsson, 2013), a reduction in behavioural plasticity can be beneficial for mate acquisition and intra‐individual competition (Munson et al., 2020), especially in species where males have to compete for both mating success and post‐copulatory sperm competition. Since fluoxetine exposure was found to reduce sperm quality (Section 4.1), adapting to behavioural tactics that are more consistent over time may improve male mating success (Schuett et al., 2010). Future empirical work is required to determine whether behavioural consistency (less within‐individual variation) in these populations results in higher reproductive success (Brand et al., 2024).

We also found dose‐specific effects of fluoxetine on between‐individual variation in body condition and sperm number, indicating a differential susceptibility of these traits to varying concentrations of the pollutant. High fluoxetine exposure increased between‐individual variation in body condition, while low fluoxetine exposure increased between‐individual variation in sperm number. This evidence, paired with the lack of mean effects of the pollutant on sperm number, suggests that some individuals have a higher trait variation than others, resulting in strong dose‐specific, individual‐level effects of the pollutant that is undetectable at the mean level. This result strengthens our argument that estimating individual‐level effects is critical to disentangling the overall impacts of global pollutants on wildlife. Although our random selection of males from all mesocosms likely resulted in a relatively uniform distribution of age‐related variation across treatments, if fluoxetine has any effect on male ageing, this could potentially impact our results on sperm traits (Gasparini, Devigili, & Pilastro, 2019). Future studies could explore the effects of age explicitly, perhaps by including individuals of known age or by examining age‐related changes in reproductive traits due to fluoxetine exposure over time.

### 4.3 Between‐individual correlation in behavioural, life‐history and sperm traits (pace‐of‐life syndrome)

A key finding of this study is that fluoxetine exposure altered between‐individual correlations in traits, revealing that individual‐level effects of the pollutant can impact far more than single traits, and can reshape an entire suite of correlated traits—as predicted by the revised pace‐of‐life syndrome hypothesis (Dammhahn et al., 2018). From an ecological perspective, the strengthening of the pace‐of‐life syndrome in fluoxetine‐exposed populations indicates that fluoxetine can reshape the way in which guppies adjust to their environment (Godin et al., 2022; Hämäläinen et al., 2021). For example, more active fish having lower body conditions than less active individuals suggests that, in polluted environments, individuals might require prioritising energy allocation across competing functions, and prefer allocating in energy reserves over behaviours linked to resource acquisition. This interpretation is supported by previous evidence, in which the between‐individual correlation between such traits was found to vary across animal populations adapted to different temperatures in damselfly (Tüzün & Stoks, 2022), predator pressures in lemon shark (Dhellemmes et al., 2021) or both temperature regimes and predator pressure in mosquitofish (Polverino et al., 2018). Similarly, the negative correlation between gonopodium size and sperm vitality, and the disruption of the positive association between gonopodium size and sperm velocity, suggests that fluoxetine exposure triggers a trade‐off between allocations in pre‐ and post‐copulatory traits, whereby longer gonopodia might confer a reproductive advantage to individuals when maintenance of sperm quality is costly. Our results reveal that the consequences of pollutants are broader than previously known, with fluoxetine altering the way in which individuals allocate their energy to adapt to changing environments.

Likewise, the disruption of other intra‐specific correlations, such as the negative correlation between activity and gonopodium size, further advocates the potential for fluoxetine to alter the trade‐offs between pace‐of‐life syndrome traits. Individual allocation in activity and sexual ornaments can be negatively associated due to the differential allocation in dimorphic life‐history strategy, as observed in killifish (Sowersby et al., 2022) and in blue tits (López‐Idiáquez et al., 2023). As fluoxetine is a selective serotonin reuptake inhibitor and is known to alter the energy balance of individuals, these findings suggest that environmental stressors may interfere with fundamental trade‐offs, contributing to shifts in individual‐level trait associations. From an evolutionary standpoint, the trade‐offs observed, especially in reproductive traits, suggest potential shifts in individual‐level tactics that may impact the long‐term evolutionary trajectories of these populations.

We found that more active individuals also exhibited higher risk‐taking behaviour (i.e. less refuge use), irrespective of pollutant exposure, suggesting a consistent behavioural syndrome between these traits (Sih et al., 2004). However, the strength of this relationship decreased as fluoxetine concentrations increased, indicating that pollution might play a role in disrupting the natural correlation between these behaviours—as also observed in mosquitofish populations adapted to other ecological stressors (Polverino et al., 2018). Indeed, stressors such as pollutants could affect the links between behavioural traits, leading to syndrome disruption or reinforcement, with important consequences for behavioural evolutionary trajectories (Jacquin et al., 2020).

More broadly, it is well established that chemical pollutants can exert strong selection pressures underpinning rapid evolutionary responses (Saaristo et al., 2018). Whether this is also true of the changes observed as a result of long‐term fluoxetine exposure—or of exposure to pharmaceutical pollutants more generally—remains to be investigated. Previous work on guppies has shown that differences in environmental conditions can result in heritable genetic changes in as little as four generations (Dargent et al., 2013). Given that fish in this study have been maintained in their respective exposure set‐ups for up to 15 overlapping generations, it is certainly conceivable that the differences observed between fluoxetine exposed and unexposed fish could reflect underlying evolutionary responses. This can be investigated experimentally by testing for the persistence of these differences after mesocosm populations have been returned to common garden conditions (i.e. free from fluoxetine) for several generations to disentangle heritable genetic differences from phenotypic plasticity or maternal effects (sensu Dargent et al., 2013; Reznick et al., 1990).

## 5 CONCLUSIONS

Our study provides valuable insights into the complex and interconnected responses of wild guppies to chronic exposure to a widespread pharmaceutical pollutant. The dose‐specific effects at the population (i.e. mean) and individual level indicate that the impact of pollutants goes beyond simple, average responses and influences specific traits and their correlations. The observed disruption in between‐individual trait correlations and pace‐of‐life syndrome suggests that fluoxetine can alter fundamental trade‐offs between behaviour, life‐history and reproduction. These findings have broader implications for understanding the adaptive capacity of wildlife facing environmental challenges. Considering the potential constraints on phenotypic variation and altered trait associations, our study highlights the need for a comprehensive and holistic approach to assessing the ecological and evolutionary consequences of pharmaceutical pollutants in aquatic ecosystems.

## AUTHOR CONTRIBUTIONS

Upama Aich, Giovanni Polverino and Bob B. M. Wong conceived the idea; Upama Aich and Giovanni Polverino designed methodology; Upama Aich, Giovanni Polverino, Farin Yazdan Parast, Gabriela C. Melo, Hung Tan, James Howells and Reza Nosrati collected the data; Upama Aich analysed the data; Upama Aich and Giovanni Polverino led the writing of the manuscript. All the authors contributed critically to the drafts and gave final approval for publication.

## CONFLICT OF INTEREST STATEMENT

The authors declare no competing interests.

## Supporting information


**Table S1.** Average fluoxetine concentration, ng/L with standard error per mesocosm population (three treatments, four replicate populations per treatment) where guppies were housed during the period of pollution exposure.
**Table S2.** Output from Bayesian multivariate mixed‐effects model investigating the effects of different fluoxetine treatments on male behaviour, life‐history and reproductive traits, including activity (distance moved, in cm), refuge use (in seconds), body condition (scaled mass‐index, in g), colouration (total proportion of orange and black accounting for body size), gonopodium size (in mm), sperm vitality (proportion of live vs. dead sperm), sperm number (total number of sperm) and sperm velocity (curvilinear velocity [VCL] in μm/s).
**Table S3.** Output from Bayesian multivariate mixed‐effects model with flat prior investigating the effects of different fluoxetine treatments on male behaviour, life‐history and reproductive traits, including activity (distance moved, in cm), refuge use (in seconds), body condition (scaled mass‐index, in g), colouration (total proportion of orange and black accounting for body size), gonopodium size (in mm), sperm vitality (proportion of live vs. dead sperm), sperm number (total number of sperm) and sperm velocity (curvilinear velocity [VCL] in μm/s).
**Table S4.** The effect size of magnitude difference in between‐individual variance estimates (ΔVA) with 89% and 95% credible intervals in activity (distance moved, in cm), refuge use (in seconds), body condition (scaled mass‐index, in g), colouration (total proportion of orange and black colouration accounting for body size), gonopodium size, in mm, sperm vitality (proportion of live vs. dead sperm) sperm count (total number of sperm) and sperm velocity (curvilinear velocity [VCL]) in males exposed to different fluoxetine treatments.
**Table S5.** The effect size of the magnitude difference in within‐individual variance estimates (ΔVW) estimates with 89% and 95% credible intervals in activity (distance moved, in cm) and refuge use (in seconds) in males exposed to different fluoxetine treatments.
**Table S6.** Between‐individual correlation estimates in behavioural, life‐history, and sperm traits across the exposure treatments (control, low, and high fluoxetine).
**Figure S1.** Between‐individual correlation in (a) behaviour, (b) life‐history, and (c) sperm traits of males.

## Data Availability

Data and code are available in Figshare: https://doi.org/10.6084/m9.figshare.24681066.v2 (Aich et al., 2024).
