# Supplementary material for: Long‐term effects of widespread pharmaceutical pollution on trade‐offs between behavioural, life‐history and reproductive traits in fish
Source: J Anim Ecol. 2024 Aug 26;94(3):340–55. doi: 10.1111/1365-2656.14152 (PMC11880659; doi:10.1111/1365-2656.14152)
Supplement: Supplementary file 1 — Table S1. Average fluoxetine concentration, ng/L with standard error per mesocosm population (three treatments, four replicate populations per treatment) where guppies were housed during the period of pollution exposure. Table S2. Output from Bayesian multivariate mixed‐effects model investigating the effects of different fluoxetine treatments on male behaviour, life‐history and reproductive traits, including activity (distance moved, in cm), refuge use (in seconds), body condition (scaled mass‐index, in g), colouration (total proportion of orange and black accounting for body size), gonopodium size (in mm), sperm vitality (proportion of live vs. dead sperm), sperm number (total number of sperm) and sperm velocity (curvilinear velocity [VCL] in μm/s). Table S3. Output from Bayesian multivariate mixed‐effects model with flat prior investigating the effects of different fluoxetine treatments on male behaviour, life‐history and reproductive traits, including activity (distance moved, in cm), refuge use (in seconds), body condition (scaled mass‐index, in g), colouration (total proportion of orange and black accounting for body size), gonopodium size (in mm), sperm vitality (proportion of live vs. dead sperm), sperm number (total number of sperm) and sperm velocity (curvilinear velocity [VCL] in μm/s). Table S4. The effect size of magnitude difference in between‐individual variance estimates (ΔVA) with 89% and 95% credible intervals in activity (distance moved, in cm), refuge use (in seconds), body condition (scaled mass‐index, in g), colouration (total proportion of orange and black colouration accounting for body size), gonopodium size, in mm, sperm vitality (proportion of live vs. dead sperm) sperm count (total number of sperm) and sperm velocity (curvilinear velocity [VCL]) in males exposed to different fluoxetine treatments. Table S5. The effect size of the magnitude difference in within‐individual variance estimates (ΔVW) estimates with 89% and 95% credible interva [file JANE-94-340-s001.docx]

**Long-term effects of widespread pharmaceutical pollution on trade-offs between behaviour, life-history, and reproductive traits in fish**

**Supporting Information**

**Glossary**

1. Among or Between-individual variance: This refers to the variation observed between different individuals within a population for a specific trait or characteristic. It represents the differences in trait values among individuals within a group or treatment. For instance, individuals within a treatment group may exhibit distinct levels of a trait, such as behaviour or morphology, contributing to between-individual variance.
2. Behavioural plasticity: Broadly defined as the ability to reversibly alter one’s behaviour in response to external or internal environmental change.
3. Variation at an individual level: This encompasses changes or differences in traits observed within the same individual organism. It includes within-individual variation, such as fluctuations in behaviour or physiological responses within an individual over time, as well as between-individual variance, which refers to differences in trait values among individuals within a population or treatment group. Understanding variation at an individual level is crucial for deciphering the complexities of trait expression and responses to environmental stimuli.
4. Within-individual variation: This refers to the variation observed in traits or characteristics within the same individual over time or under different conditions. For example, changes in behaviour, physiology, or morphology that occur within an individual organism across different days, contexts, or experimental treatments.

**Table S1.** Average fluoxetine concentration, ng/L with standard error per mesocosm population (3 treatments, 4 replicate populations per treatment) where guppies were housed during the period of pollution exposure. Each mesocosm tank was sampled monthly for water analysis during the study period (n=59). Water analysis was performed using gas chromatography–tandem mass spectrometry with a minimum detection limit of 2 ng/L (see Tan et al., 2020 for methodological details).

| Treatments | Mesocosm | Mean | SE |
| --- | --- | --- | --- |
| Control | C1 | <2 | 0 |
|  | C2 | <2 | 0 |
|  | C3 | <2 | 0 |
|  | C4 | <2 | 0.156 |
| Low | L1 | 36.498 | 5.113 |
|  | L2 | 27.059 | 2.570 |
|  | L3 | 29.756 | 3.073 |
|  | L4 | 32.812 | 4.244 |
| High | H1 | 326.031 | 34.463 |
|  | H2 | 268.062 | 31.826 |
|  | H3 | 328.604 | 37.857 |
|  | H4 | 341.832 | 42.814 |

**Table S2.** Output from Bayesian multivariate mixed-effects model investigating the effects of different fluoxetine treatments on male behaviour, life-history and reproductive traits, including activity (distance moved, in cm), refuge use (in seconds), body condition (scaled mass-index, in g), colouration (total proportion of orange and black accounting for body size), gonopodium size (in mm), sperm vitality (proportion of live vs dead sperm), sperm number (total number of sperm) and sperm velocity (Curvilinear Velocity-VCL in μm/s). Bold estimates with 89% and 95% credible intervals indicate fixed effects and intercept values that differ from or are very close to zero. Treatment (control) is the reference category for fixed effects. Random effects are presented in standard deviation (SD) units.

| Traits | Variables | Estimate | 89% CIs | 95% CIs |
| --- | --- | --- | --- | --- |
|  |  |  |  |  |
| Activity | Distance moved: (Intercept) | **0.36** | **(0.11, 0.61)** | **(0.04, 0.67)** |
|  | Distance moved: Treatment (High Fluoxetine) | -0.12 | (-0.45, 0.22) | (-0.53, 0.3) |
|  | Distance moved: Treatment (Low Fluoxetine) | -0.28 | (-0.6, 0.04) | (-0.68, 0.12) |
|  | Distance moved: trial (left-centred) | **-0.22** | **(-0.29, -0.16)** | **(-0.31, -0.14)** |
| Refuge use | In refuge: (Intercept) | **-0.35** | **(-0.57, -0.12)** | **(-0.62, -0.06)** |
|  | In refuge: Treatment (High Fluoxetine) | 0.18 | (-0.11, 0.47) | (-0.18, 0.54) |
|  | In refuge: Treatment (Low Fluoxetine) | 0.26 | (-0.04, 0.55) | (-0.11, 0.63) |
|  | In refuge: trial (left-centred) | **0.19** | **(0.11, 0.27)** | **(0.09, 0.29)** |
| Body condition | Scaled mass index: (Intercept) | 0 | (-0.32, 0.31) | (-0.4, 0.42) |
|  | Scaled mass index: Treatment (High Fluoxetine) | **0.48** | **(0.01, 0.96)** | (-0.12, 1.08) |
|  | Scaled mass index: Treatment (Low Fluoxetine) | **-0.52** | **(-0.97, -0.06)** | (-1.1, 0.06) |
| Colouration | Colouration: (Intercept) | -0.19 | (-0.51, 0.12) | (-0.59, 0.21) |
|  | Colouration: Treatment (High Fluoxetine) | 0.25 | (-0.22, 0.72) | (-0.36, 0.85) |
|  | Colouration: Treatment (Low Fluoxetine) | 0.33 | (-0.15, 0.79) | (-0.28, 0.91) |
| Gonopodium length | Gonopodium length: (Intercept) | -0.34 | (-0.69, 0.01) | (-0.78, 0.11) |
|  | Gonopodium length: Treatment (High Fluoxetine) | 0.42 | (-0.08, 0.91) | (-0.22, 1.05) |
|  | Gonopodium length: Treatment (Low Fluoxetine) | **0.61** | **(0.09, 1.12)** | (-0.06, 1.27) |
| Sperm vitality | Vitality percentage: (Intercept) | -0.14 | (-0.44, 0.17) | (-0.52, 0.25) |
|  | Vitality percentage: Treatment (High Fluoxetine) | 0.28 | (-0.14, 0.71) | (-0.24, 0.8) |
|  | Vitality percentage: Treatment (Low Fluoxetine) | 0.12 | (-0.31, 0.55) | (-0.42, 0.68) |
| Sperm number | Sperm count: (Intercept) | -0.15 | (-0.47, 0.18) | (-0.56, 0.27) |
|  | Sperm count: Treatment (High Fluoxetine) | 0.1 | (-0.34, 0.53) | (-0.46, 0.67) |
|  | Sperm count: Treatment (Low Fluoxetine) | 0.36 | (-0.16, 0.89) | (-0.28, 1.03) |
| Sperm velocity | VCL (Intercept) | 0.26 | (-0.02, 0.56) | (-0.1, 0.63) |
|  | VCL Treatment (High Fluoxetine) | -0.35 | (-0.76, 0.05) | (-0.87, 0.15) |
|  | VCL Treatment (Low Fluoxetine) | **-0.45** | **(-0.88, -0.02)** | (-1, 0.09) |
| Random intercept | |  |  |  |
| Male ID | Distance moved: Treatment (Control) | **0.71** | **(0.55, 0.89)** | **(0.51, 0.93)** |
|  | Distance moved: Treatment (High Fluoxetine) | **0.72** | **(0.58, 0.89)** | **(0.55, 0.93)** |
|  | Distance moved: Treatment (Low Fluoxetine) | **0.6** | **(0.47, 0.75)** | **(0.44, 0.79)** |
|  | In refuge: Treatment (Control) | **0.53** | **(0.36, 0.71)** | **(0.31, 0.76)** |
|  | In refuge: Treatment (High Fluoxetine) | **0.54** | **(0.37, 0.72)** | **(0.33, 0.77)** |
|  | In refuge: Treatment (Low Fluoxetine) | **0.61** | **(0.45, 0.77)** | **(0.42, 0.82)** |
|  | Scaled mass index: Treatment (Control) | **0.8** | **(0.67, 0.97)** | **(0.65, 1.01)** |
|  | Scaled mass index: Treatment (High Fluoxetine) | **1.09** | **(0.91, 1.29)** | **(0.88, 1.35)** |
|  | Scaled mass index: Treatment (Low Fluoxetine) | **0.86** | **(0.71, 1.03)** | **(0.69, 1.08)** |
|  | Colouration: Treatment (Control) | **0.91** | **(0.75, 1.09)** | **(0.72, 1.14)** |
|  | Colouration: Treatment (High Fluoxetine) | **1.09** | **(0.92, 1.3)** | **(0.89, 1.36)** |
|  | Colouration: Treatment (Low Fluoxetine) | **1.09** | **(0.91, 1.3)** | **(0.88, 1.35)** |
|  | Gonopodium length: Treatment (Control) | **0.95** | **(0.8, 1.13)** | **(0.77, 1.18)** |
|  | Gonopodium length: Treatment (High Fluoxetine) | **0.92** | **(0.77, 1.09)** | **(0.74, 1.14)** |
|  | Gonopodium length: Treatment (Low Fluoxetine) | **1.09** | **(0.91, 1.3)** | **(0.87, 1.36)** |
|  | Vitality percentage: Treatment (Control) | **1.09** | **(0.91, 1.3)** | **(0.88, 1.35)** |
|  | Vitality percentage: Treatment (High Fluoxetine) | **0.98** | **(0.83, 1.2)** | **(0.8, 1.22)** |
|  | Vitality percentage: Treatment (Low Fluoxetine) | **1.07** | **(0.89, 1.28)** | **(0.86, 1.34)** |
|  | Sperm count: Treatment (Control) | **0.89** | **(0.74, 1.07)** | **(0.72, 1.11)** |
|  | Sperm count: Treatment (High Fluoxetine) | **0.77** | **(0.65, 0.92)** | **(0.62, 0.96)** |
|  | Sperm count: Treatment (Low Fluoxetine) | **1.31** | **(1.1, 1.56)** | **(1.06, 1.63)** |
|  | VCL Treatment (Control) | **0.98** | **(0.82, 1.18)** | **(0.79, 1.23)** |
|  | VCL Treatment (High Fluoxetine) | **1** | **(0.84, 1.18)** | **(0.81, 1.24)** |
|  | VCL Treatment (Low Fluoxetine) | **1.12** | **(0.94, 1.33)** | **(0.9, 1.4)** |
| Mesocosm | Distance moved: (Intercept) | **0.14** | **(0.01, 0.31)** | **(0.01, 0.37)** |
|  | In refuge: (Intercept) | **0.12** | **(0.01, 0.27)** | **(0, 0.32)** |
|  | Scaled mass index: (Intercept) | **0.28** | **(0.05, 0.56)** | **(0.02, 0.64)** |
|  | Colouration: (Intercept) | **0.26** | **(0.04, 0.53)** | **(0.01, 0.62)** |
|  | Gonopodium length: (Intercept) | **0.31** | **(0.07, 0.58)** | **(0.03, 0.67)** |
|  | Vitality percentage: (Intercept) | **0.17** | **(0.01, 0.4)** | **(0.01, 0.48)** |
|  | Sperm count: (Intercept) | **0.28** | **(0.05, 0.56)** | **(0.02, 0.64)** |
|  | VCL (Intercept) | **0.17** | **(0.02, 0.4)** | **(0.01, 0.48)** |
| Residual variances | |  |  |  |
|  | Distance moved: Treatment (Control) | **-0.22** | **(-0.34, -0.1)** | **(-0.36, -0.07)** |
|  | Distance moved: Treatment (High Fluoxetine) | **-0.4** | **(-0.51, -0.28)** | **(-0.54, -0.25)** |
|  | Distance moved: Treatment (Low Fluoxetine) | **-0.54** | **(-0.66, -0.41)** | **(-0.69, -0.38)** |
|  | In refuge: Treatment (Control) | **-0.12** | **(-0.24, 0)** | **(-0.26, 0.03)** |
|  | In refuge: Treatment (High Fluoxetine) | **-0.21** | **(-0.32, -0.09)** | **(-0.35, -0.06)** |
|  | In refuge: Treatment (Low Fluoxetine) | **-0.32** | **(-0.44, -0.2)** | **(-0.47, -0.17)** |

**Table S3.** Output from Bayesian multivariate mixed-effects model with flat prior investigating the effects of different fluoxetine treatments on male behaviour, life-history and reproductive traits, including activity (distance moved, in cm), refuge use (in seconds), body condition (scaled mass-index, in g), colouration (total proportion of orange and black accounting for body size), gonopodium size (in mm), sperm vitality (proportion of live vs dead sperm), sperm number (total number of sperm) and sperm velocity (Curvilinear Velocity-VCL in μm/s). Bold estimates with 89% and 95% credible intervals indicate fixed effects and intercept values that differ from or are very close to zero. Treatment (control) is the reference category for fixed effects. Random effects are presented in standard deviation (SD) units.

| Traits | Variable | Estimate | 89% CI | 95% CIs |
| --- | --- | --- | --- | --- |
|  |  |  |  |  |
| Activity | Distance moved: (Intercept) | **0.36** | **(0.11, 0.62)** | **(0.04, 0.68)** |
|  | Distance moved: Treatment (High Fluoxetine) | -0.12 | (-0.46, 0.23) | (-0.54, 0.31) |
|  | Distance moved: Treatment (Low Fluoxetine) | -0.28 | (-0.61, 0.04) | (-0.69, 0.13) |
|  | Distance moved: trial (left-centred) | **-0.22** | **(-0.29, -0.16)** | **(-0.3, -0.14)** |
| Refuge use | In refuge: (Intercept) | **-0.34** | **(-0.57, -0.11)** | **(-0.63, -0.06)** |
|  | In refuge: Treatment (High Fluoxetine) | 0.18 | (-0.12, 0.47) | (-0.2, 0.55) |
|  | In refuge: Treatment (Low Fluoxetine) | 0.25 | (-0.05, 0.56) | (-0.13, 0.63) |
|  | In refuge: trial (left-centred) | **0.19** | **(0.11, 0.27)** | **(0.09, 0.29)** |
| Body condition | Scaled mass index: (Intercept) | 0 | (-0.33, 0.32) | (-0.42, 0.41) |
|  | Scaled mass index: Treatment (High Fluoxetine) | **0.49** | **(0.04, 0.99)** | (-0.15, 1.14) |
|  | Scaled mass index: Treatment (Low Fluoxetine) | **-0.51** | **(-0.98, -0.05)** | (-1.11, 0.09) |
| Colouration | Colouration: (Intercept) | -0.2 | (-0.52, 0.14) | (-0.61, 0.23) |
|  | Colouration: Treatment (High Fluoxetine) | 0.25 | (-0.24, 0.74) | (-0.39, 0.87) |
|  | Colouration: Treatment (Low Fluoxetine) | 0.33 | (-0.17, 0.82) | (-0.31, 0.95) |
| Gonopodium length | Gonopodium length: (Intercept) | -0.35 | (-0.72, 0.01) | (-0.83, 0.12) |
|  | Gonopodium length: Treatment (High Fluoxetine) | 0.43 | (-0.08, 0.93) | (-0.2, 1.08) |
|  | Gonopodium length: Treatment (Low Fluoxetine) | **0.61** | **(0.08, 1.15)** | (-0.07, 1.29) |
| Sperm vitality | Vitality percentage: (Intercept) | -0.14 | (-0.46, 0.17) | (-0.55, 0.25) |
|  | Vitality percentage: Treatment (High Fluoxetine) | 0.28 | (-0.14, 0.7) | (-0.25, 0.83) |
|  | Vitality percentage: Treatment (Low Fluoxetine) | 0.13 | (-0.31, 0.57) | (-0.41, 0.69) |
| Sperm number | Sperm count: (Intercept) | -0.14 | (-0.48, 0.18) | (-0.59, 0.3) |
|  | Sperm count: Treatment (High Fluoxetine) | 0.1 | (-0.38, 0.56) | (-0.51, 0.69) |
|  | Sperm count: Treatment (Low Fluoxetine) | 0.36 | (-0.18, 0.9) | (-0.33, 1.05) |
| Sperm velocity | VCL (Intercept) | 0.27 | (-0.03, 0.57) | (-0.12, 0.65) |
|  | VCL Treatment (High Fluoxetine) | -0.36 | (-0.78, 0.06) | (-0.91, 0.16) |
|  | VCL Treatment (Low Fluoxetine) | **-0.45** | **(-0.9, -0.02)** | (-1.01, 0.09) |
| Random intercepts | |  |  |  |
| Male ID | Distance moved: Treatment (Control) | **0.72** | **(0.56, 0.91)** | **(0.52, 0.96)** |
|  | Distance moved: Treatment (High Fluoxetine) | **0.73** | **(0.58, 0.9)** | **(0.56, 0.95)** |
|  | Distance moved: Treatment (Low Fluoxetine) | **0.61** | **(0.48, 0.76)** | **(0.45, 0.8)** |
|  | In refuge: Treatment (Control) | **0.55** | **(0.37, 0.73)** | **(0.33, 0.78)** |
|  | In refuge: Treatment (High Fluoxetine) | **0.55** | **(0.38, 0.73)** | **(0.33, 0.77)** |
|  | In refuge: Treatment (Low Fluoxetine) | **0.62** | **(0.46, 0.79)** | **(0.43, 0.84)** |
|  | Scaled mass index: Treatment (Control) | **0.81** | **(0.68, 0.97)** | **(0.65, 1.02)** |
|  | Scaled mass index: Treatment (High Fluoxetine) | **1.1** | **(0.91, 1.31)** | **(0.88, 1.37)** |
|  | Scaled mass index: Treatment (Low Fluoxetine) | **0.86** | **(0.72, 1.04)** | **(0.69, 1.09)** |
|  | Colouration: Treatment (Control) | **0.91** | **(0.76, 1.08)** | **(0.73, 1.15)** |
|  | Colouration: Treatment (High Fluoxetine) | **1.1** | **(0.93, 1.31)** | **(0.89, 1.37)** |
|  | Colouration: Treatment (Low Fluoxetine) | **1.1** | **(0.91, 1.31)** | **(0.88, 1.38)** |
|  | Gonopodium length: Treatment (Control) | **0.96** | **(0.81, 1.15)** | **(0.77, 1.2)** |
|  | Gonopodium length: Treatment (High Fluoxetine) | **0.92** | **(0.77, 1.09)** | **(0.75, 1.15)** |
|  | Gonopodium length: Treatment (Low Fluoxetine) | **1.1** | **(0.92, 1.32)** | **(0.89, 1.39)** |
|  | Vitality percentage: Treatment (Control) | **1.1** | **(0.91, 1.32)** | **(0.88, 1.38)** |
|  | Vitality percentage: Treatment (High Fluoxetine) | **0.99** | **(0.84, 1.18)** | **(0.81, 1.23)** |
|  | Vitality percentage: Treatment (Low Fluoxetine) | **1.08** | **(0.9, 1.3)** | **(0.87, 1.36)** |
|  | Sperm count: Treatment (Control) | **0.89** | **(0.75, 1.08)** | **(0.72, 1.13)** |
|  | Sperm count: Treatment (High Fluoxetine) | **0.78** | **(0.65, 0.93)** | **(0.63, 0.97)** |
|  | Sperm count: Treatment (Low Fluoxetine) | **1.33** | **(1.11, 1.6)** | **(1.08, 1.67)** |
|  | VCL Treatment (Control) | **0.99** | **(0.83, 1.18)** | **(0.8, 1.23)** |
|  | VCL Treatment (High Fluoxetine) | **1.01** | **(0.85, 1.2)** | **(0.82, 1.26)** |
|  | VCL Treatment (Low Fluoxetine) | **1.13** | **(0.94, 1.35)** | **(0.91, 1.41)** |
| Mesocosm | Distance moved: (Intercept) | **0.15** | **(0.01, 0.34)** | **(0.01, 0.4)** |
|  | In refuge: (Intercept) | **0.12** | **(0.01, 0.29)** | **(0, 0.34)** |
|  | Scaled mass index: (Intercept) | **0.3** | **(0.06, 0.59)** | **(0.03, 0.69)** |
|  | Colouration: (Intercept) | **0.28** | **(0.05, 0.6)** | **(0.02, 0.67)** |
|  | Gonopodium length: (Intercept) | **0.32** | **(0.08, 0.59)** | **(0.04, 0.71)** |
|  | Vitality percentage: (Intercept) | **0.18** | **(0.02, 0.43)** | **(0.01, 0.51)** |
|  | Sperm count: (Intercept) | **0.3** | **(0.06, 0.53)** | **(0.03, 0.7)** |
|  | VCL (Intercept) | **0.2** | **(0.02, 0.43)** | **(0.01, 0.51)** |
| Residual variances | |  |  |  |
|  | Distance moved: Treatment (Control) | **-0.22** | **(-0.34, -0.1)** | **(-0.37, -0.07)** |
|  | Distance moved: Treatment (High Fluoxetine) | **-0.4** | **(-0.51, -0.28)** | **(-0.53, -0.25)** |
|  | Distance moved: Treatment (Low Fluoxetine) | **-0.54** | **(-0.66, -0.42)** | **(-0.69, -0.39)** |
|  | In refuge: Treatment (Control) | **-0.13** | **(-0.24, -0.01)** | **(-0.27, 0.03)** |
|  | In refuge: Treatment (High Fluoxetine) | **-0.21** | **(-0.32, -0.09)** | **(-0.35, -0.06)** |
|  | In refuge: Treatment (Low Fluoxetine) | **-0.33** | **(-0.44, -0.19)** | **(-0.47, -0.17)** |

**Table S4.** The effect size of magnitude difference in between-individual variance estimates (ΔVA) with 89% and 95% credible intervals in activity (distance moved, in cm), refuge use (in seconds), body condition (scaled mass-index, in g), colouration (total proportion of orange and black colouration accounting for body size), gonopodium size, in mm, sperm vitality (proportion of live vs dead sperm) sperm count (total number of sperm) and sperm velocity (Curvilinear Velocity-VCL) in males exposed to different fluoxetine treatments. Bold values with 89% and 95% credible intervals indicate estimates that differ from or are very close to zero.

| Activity |  |  |  |
| --- | --- | --- | --- |
| Contrast | Distance moved | 89% CIs | 95% CIs |
| Control-High | -0.016 | (-0.339, 0.334) | (-0.444, 0.398) |
| Control-Low | 0.143 | (-0.15, 0.454) | (-0.226, 0.527) |
| High-Low | 0.159 | (-0.126, 0.448) | (-0.192, 0.526) |
| Refuge use |  |  |  |
| Contrast | Refuge use | 89% CIs | 95% CIs |
| Control-High | -0.006 | (-0.269, 0.271) | (-0.34, 0.339) |
| Control-Low | -0.08 | (-0.356, 0.204) | (-0.433, 0.269) |
| High-Low | -0.074 | (-0.356, 0.195) | (-0.421, 0.282) |
| Body condition | |  |  |
| Contrast | Scaled mass index | 89% CIs | 95% CIs |
| Control-High | **-0.537** | **(-1.025, -0.072)** | (-1.19, 0.03) |
| Control-Low | -0.09 | (-0.452, 0.288) | (-0.572, 0.362) |
| High-Low | 0.45 | (-0.028, 0.966) | (-0.176, 1.086) |
| Colouration |  |  |  |
| Contrast | Total colour | 89% CIs | 95% CIs |
| Control-High | -0.379 | (-0.897, 0.147) | (-1.025, 0.305) |
| Control-Low | -0.365 | (-0.909, 0.158) | (-1.077, 0.278) |
| High-Low | 0.014 | (-0.626, 0.593) | (-0.757, 0.793) |
| Gonopodium size | |  |  |
| Contrast | Gonopodium size | 89% CIs | 95% CIs |
| Control-High | 0.064 | (-0.364, 0.524) | (-0.503, 0.624) |
| Control-Low | -0.282 | (-0.835, 0.3) | (-0.99, 0.376) |
| High-Low | -0.347 | (-0.855, 0.136) | (-1.06, 0.275) |
| Sperm velocity | |  |  |
| Contrast | Curvilinear Velocity | 89% CIs | 95% CIs |
| Control-High | -0.029 | (-0.55, 0.449) | (-0.66, 0.608) |
| Control-Low | -0.289 | (-0.85, 0.3) | (-1.058, 0.396) |
| High-Low | -0.26 | (-0.833, 0.313) | (-1.016, 0.457) |
| Sperm vitality | |  |  |
| Contrast | Vitality | 89% CIs | 95% CIs |
| Control-High | 0.217 | (-0.331, 0.758) | (-0.466, 0.918) |
| Control-Low | 0.031 | (-0.592, 0.629) | (-0.748, 0.804) |
| High-Low | -0.186 | (-0.777, 0.329) | (-0.899, 0.499) |
| Sperm Number | |  |  |
| Contrast | Total sperm count | 89% CIs | 95% CIs |
| Control-High | 0.198 | (-0.17, 0.557) | (-0.25, 0.663) |
| Control-Low | **-0.945** | **(-1.576, -0.232)** | **(-1.798, -0.115)** |
| High-Low | **-1.143** | **(-1.771, -0.503)** | **(-1.978, -0.389)** |

**Table S5.** The effect size of the magnitude difference in within-individual variance estimates (ΔVW) estimates with 89% and 95% credible intervals in activity (distance moved, in cm) and refuge use (in seconds) in males exposed to different fluoxetine treatments. Bold values with 89% and 95% credible intervals indicate estimates that differ from or are very close to zero.

| Activity |  |  |  |
| --- | --- | --- | --- |
| Contrast | Distance moved | 89% CIs | 95% CIs |
| Control-High | **0.193** | **(0.004, 0.375)** | (-0.048, 0.424) |
| Control-Low | **0.307** | **(0.124, 0.48)** | **(0.088, 0.536)** |
| High-Low | 0.114 | (-0.025, 0.252) | (-0.049, 0.296) |
| Refuge use |  |  |  |
| Contrast | Refuge use | 89% CIs | 95% CIs |
| Control-High | 0.119 | (-0.147, 0.375) | (-0.193, 0.43) |
| Control-Low | **0.259** | **(0.022, 0.486)** | (-0.011, 0.562) |
| High-Low | 0.14 | (-0.06, 0.358) | (-0.125, 0.39) |

**Table S6.** Between-individual correlation estimates in behavioural, life-history, and sperm traits across the exposure treatments (control, low, and high fluoxetine). Bold values with 89% and 95% credible intervals indicate estimates that differ from or are very close to zero.

| Correlation Pairs | Treatments | Estimate | 89% CIs | 95% CIs |
| --- | --- | --- | --- | --- |
| Behaviour |  |  |  |  |
| Activity, Refuge use | Control | **-0.431** | **(-0.643, -0.181)** | **(-0.681, -0.12)** |
|  | Low | **-0.367** | **(-0.576, -0.127)** | **(-0.613, -0.072)** |
|  | High | **-0.357** | **(-0.57, -0.117)** | **(-0.61, -0.061)** |
| Behaviour, life history traits |  |  |  |  |
| Activity, body condition | Control | -0.115 | (-0.333, 0.122) | (-0.38, 0.172) |
|  | Low | *-0.154* | (-0.362, 0.063) | (-0.41, 0.114) |
|  | High | **-0.276** | **(-0.464, -0.065)** | **(-0.502, -0.021)** |
| Activity, colouration | Control | 0.093 | (-0.132, 0.315) | (-0.186, 0.358) |
|  | Low | -0.02 | (-0.233, 0.195) | (-0.281, 0.245) |
|  | High | -0.103 | (-0.304, 0.114) | (-0.34, 0.167) |
| Activity, gonopodium size | Control | **-0.256** | **(-0.461, -0.027)** | (-0.502, 0.024) |
|  | Low | 0.115 | (-0.103, 0.325) | (-0.154, 0.37) |
|  | High | -0.097 | (-0.299, 0.116) | (-0.345, 0.161) |
| Refuge-use, body condition | Control | 0.052 | (-0.19, 0.289) | (-0.24, 0.341) |
|  | Low | 0.085 | (-0.15, 0.304) | (-0.196, 0.35) |
|  | High | -0.062 | (-0.29, 0.172) | (-0.341, 0.219) |
| Refuge-use, colouration | Control | -0.081 | (-0.32, 0.161) | (-0.363, 0.215) |
|  | Low | 0.08 | (-0.153, 0.308) | (-0.206, 0.357) |
|  | High | 0.094 | (-0.14, 0.321) | (-0.182, 0.368) |
| Refuge-use, gonopodium size | Control | *0.183* | (-0.059, 0.408) | (-0.094, 0.454) |
|  | Low | -0.021 | (-0.251, 0.203) | (-0.298, 0.254) |
|  | High | 0.08 | (-0.157, 0.306) | (-0.214, 0.351) |
| Behaviour, sperm traits |  |  |  |  |
| Activity, sperm vitality | Control | 0.078 | (-0.15, 0.298) | (-0.197, 0.348) |
|  | Low | -0.05 | (-0.26, 0.176) | (-0.308, 0.223) |
|  | High | 0.08 | (-0.131, 0.286) | (-0.177, 0.33) |
| Activity, sperm count | Control | -0.114 | (-0.331, 0.114) | (-0.379, 0.164) |
|  | Low | *0.134* | (-0.085, 0.34) | (-0.134, 0.381) |
|  | High | -0.119 | (-0.321, 0.091) | (-0.358, 0.141) |
| Activity, sperm velocity | Control | 0.026 | (-0.2, 0.246) | (-0.251, 0.292) |
|  | Low | *-0.128* | (-0.336, 0.094) | (-0.385, 0.147) |
|  | High | -0.07 | (-0.275, 0.14) | (-0.319, 0.186) |
| Refuge-use, sperm vitality | Control | -0.08 | (-0.31, 0.162) | (-0.366, 0.214) |
|  | Low | 0.051 | (-0.183, 0.282) | (-0.229, 0.332) |
|  | High | 0.001 | (-0.23, 0.228) | (-0.278, 0.275) |
| Refuge-use, sperm count | Control | 0.021 | (-0.216, 0.26) | (-0.265, 0.306) |
|  | Low | -0.082 | (-0.308, 0.15) | (-0.357, 0.203) |
|  | High | 0.117 | (-0.118, 0.338) | (-0.169, 0.387) |
| Refuge-use, sperm velocity | Control | 0.053 | (-0.183, 0.289) | (-0.24, 0.337) |
|  | Low | **0.229** | **(0.002, 0.439)** | (-0.053, 0.483) |
|  | High | *0.156* | (-0.08, 0.378) | (-0.136, 0.425) |
| Life-history traits |  |  |  |  |
| Body condition, colouration | Control | -0.06 | (-0.256, 0.143) | (-0.305, 0.187) |
|  | Low | 0.017 | (-0.181, 0.214) | (-0.222, 0.258) |
|  | High | *-0.171* | (-0.355, 0.024) | (-0.397, 0.066) |
| Body condition, gonopodium size | Control | 0.012 | (-0.19, 0.209) | (-0.23, 0.251) |
|  | Low | 0.055 | (-0.147, 0.25) | (-0.192, 0.295) |
|  | High | 0.007 | (-0.189, 0.203) | (-0.234, 0.246) |
| Colouration, gonopodium size | Control | 0.035 | (-0.166, 0.235) | (-0.21, 0.28) |
|  | Low | *-0.14* | (-0.325, 0.058) | (-0.371, 0.101) |
|  | High | 0.02 | (-0.18, 0.214) | (-0.22, 0.253) |
| Life-history and sperm traits |  |  |  |  |
| Body condition, sperm vitality | Control | -0.101 | (-0.297, 0.101) | (-0.336, 0.143) |
|  | Low | *0.12* | (-0.088, 0.314) | (-0.135, 0.354) |
|  | High | *-0.102* | (-0.29, 0.095) | (-0.329, 0.139) |
| Body condition, sperm count | Control | 0.025 | (-0.178, 0.222) | (-0.222, 0.265) |
|  | Low | 0.079 | (-0.117, 0.272) | (-0.173, 0.312) |
|  | High | -0.09 | (-0.282, 0.113) | (-0.324, 0.157) |
| Body condition, sperm velocity | Control | 0.061 | (-0.14, 0.26) | (-0.182, 0.298) |
|  | Low | -0.018 | (-0.214, 0.179) | (-0.254, 0.222) |
|  | High | 0.043 | (-0.151, 0.234) | (-0.192, 0.272) |
| Colouration, sperm vitality | Control | -*0.133* | (-0.322, 0.067) | (-0.364, 0.118) |
|  | Low | 0.026 | (-0.175, 0.224) | (-0.219, 0.267) |
|  | High | -*0.144* | (-0.331, 0.049) | (-0.371, 0.092) |
| Colouration, sperm count | Control | 0.052 | (-0.145, 0.251) | (-0.193, 0.295) |
|  | Low | 0.044 | (-0.152, 0.245) | (-0.196, 0.285) |
|  | High | *0.138* | (-0.059, 0.33) | (-0.107, 0.366) |
| Colouration, sperm velocity | Control | -0.116 | (-0.309, 0.088) | (-0.351, 0.132) |
|  | Low | *0.156* | (-0.04, 0.344) | (-0.093, 0.387) |
|  | High | -0.074 | (-0.264, 0.12) | (-0.305, 0.165) |
| Gonopodium size, sperm vitality | Control | -0.122 | (-0.319, 0.072) | (-0.356, 0.117) |
|  | Low | -0.03 | (-0.226, 0.176) | (-0.266, 0.224) |
|  | High | **-0.224** | **(-0.407, -0.024)** | (-0.446, 0.021) |
| Gonopodium size, sperm count | Control | -0.111 | (-0.305, 0.091) | (-0.345, 0.135) |
|  | Low | *0.1844* | (-0.015, 0.372) | (-0.065, 0.41) |
|  | High | 0.1 | (-0.099, 0.29) | (-0.144, 0.325) |
| Gonopodium size, sperm velocity | Control | **0.22** | **(0.02, 0.406)** | (-0.026, 0.441) |
|  | Low | *-0.126* | (-0.321, 0.071) | (-0.361, 0.112) |
|  | High | 0.03 | (-0.163, 0.224) | (-0.202, 0.267) |
| Sperm traits |  |  |  |  |
| Sperm vitality, sperm count | Control | 0.098 | (-0.096, 0.29) | (-0.146, 0.33) |
|  | Low | **-0.212** | **(-0.393, -0.01)** | (-0.429, 0.04) |
|  | High | 0.082 | (-0.114, 0.273) | (-0.158, 0.318) |
| Sperm vitality, sperm velocity | Control | -0.0085 | (-0.208, 0.188) | (-0.256, 0.232) |
|  | Low | -0.053 | (-0.251, 0.146) | (-0.293, 0.19) |
|  | High | -0.06 | (-0.25, 0.135) | (-0.291, 0.178) |
| Sperm count, sperm velocity | Control | 0.03 | (-0.168, 0.228) | (-0.214, 0.271) |
|  | Low | 0.005 | (-0.19, 0.203) | (-0.233, 0.245) |
|  | High | 0.128 | (-0.073, 0.317) | (-0.119, 0.359) |

**
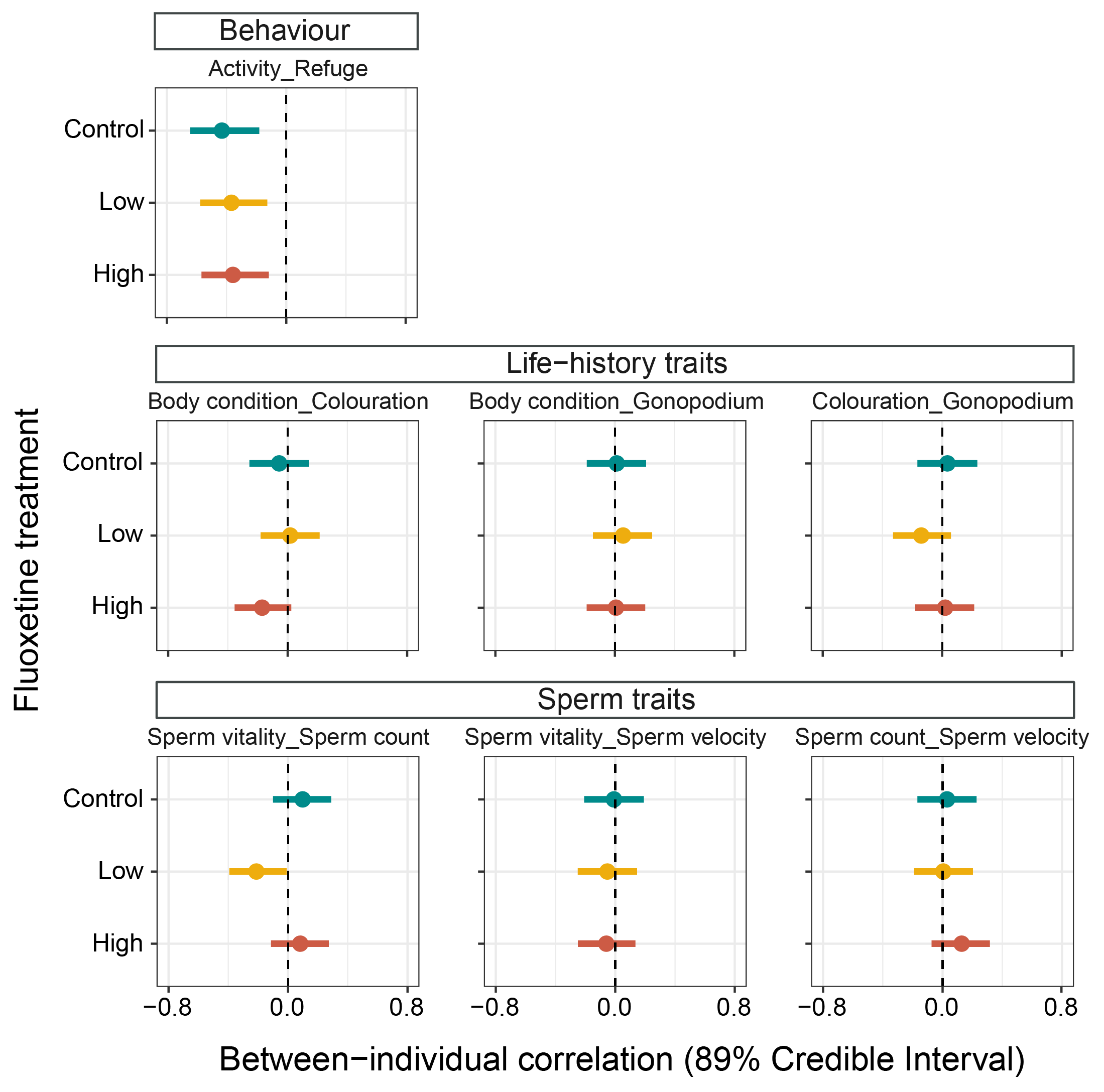
**

**a)**

**b)**

**c)**

**Fig S1.** Between-individual correlation in a) behaviour, b) life-history, and c) sperm traits of males. In each plot, filled circles with horizontal error bars denote correlational values 89% credible intervals, and the colours represent three exposure treatments (control, low fluoxetine, and high fluoxetine). The black dashed line indicates no correlation effects, while the values on the right side of the line show a positive relationship, and the ones on the left side indicate a negative relationship between traits.

**References:**

1. Tan, H., Polverino, G., Martin, J.M., Bertram, M.G., Wiles, S.C., Palacios, M.M., Bywater, C.L., White, C.R. & Wong, B.B. (2020). Chronic exposure to a pervasive pharmaceutical pollutant erodes among-individual phenotypic variation in a fish. *Environmental Pollution*, **263**, p.114450. <https://doi.org/10.1016/j.envpol.2020.114450>
